# Plant growth-promoting *Pseudomonas* strains modulate potato tuberization signalling and development

**DOI:** 10.1093/jxb/erag237

**Published:** 2026-05-20

**Authors:** Arti Mishra, Lovely Mahawar, Angeliki Tsitouri, Jasim Basheer, Benedicte Riber Albrectsen

**Affiliations:** Umeå Plant Science Centre (UPSC), Department of Plant Physiology, Umeå University, Umeå 901 87, Sweden; Department of Botany, Hansraj College, University of Delhi, Delhi 110007, India; Umeå Plant Science Centre (UPSC), Department of Plant Physiology, Umeå University, Umeå 901 87, Sweden; Umeå Plant Science Centre (UPSC), Department of Plant Physiology, Umeå University, Umeå 901 87, Sweden; Umeå Plant Science Centre (UPSC), Department of Forest Genetics and Plant Physiology, Swedish University of Agricultural Sciences, Umeå 901 83, Sweden; Umeå Plant Science Centre (UPSC), Department of Plant Physiology, Umeå University, Umeå 901 87, Sweden; Universität zu Köln, Germany

**Keywords:** metabolomic profiling, microbial consortia, plant growth-promoting rhizobacteria, plant–microbe interactions, potato (*Solanum tuberosum*), tuberization signalling

## Abstract

Plant growth-promoting rhizobacteria (PGPR) can influence plant development through hormone signalling, nutrient mobilization, and activation of defence pathways. While individual bacterial strains can enhance plant performance, microbial consortia may generate complementary or synergistic effects that remain poorly understood, particularly with respect to crop developmental signalling. Potato (*Solanum tuberosum*), the most important dicot food crop globally, represents a suitable model for investigating how beneficial microbes influence tuber development. In this study, we investigated the effects of two well-characterized PGPR strains, *Pseudomonas protegens* CHA0 and *P. simiae* WCS417, applied individually or in combination, on two potato cultivars (‘Mandel’ and ‘Désirée’) under long-day conditions. Confocal microscopy confirmed rapid root colonization by both strains within 24 h of inoculation. Metabolomic profiling of bacterial exudates revealed distinct metabolic signatures for the two strains and non-additive metabolite patterns when cultured together, suggesting metabolic interactions within the bacterial consortium. Plant responses were cultivar dependent, with bacterial treatments influencing vegetative growth and selected tuber quality traits, including starch and ascorbic acid levels. Gene expression analyses revealed strong induction of the tuberization regulator *StSP6A* in roots, with up to 5-fold increased expression following *P. protegens* and combined inoculation, accompanied by activation of jasmonic acid-related signalling pathways. Together, these results indicate that interactions between beneficial *Pseudomonas* strains can influence potato development through coordinated effects on root architecture and signalling pathways associated with tuberization and defence.

## Teaser

Dual inoculation with *Pseudomonas protegens* CHA0 and *Pseudomonas simiae* WCS417 enhances potato tuber initiation and yield via cultivar-dependent modulation of growth and defence signalling.

## Introduction

As agriculture seeks to reduce dependence on chemical inputs while maintaining productivity, integrating beneficial microorganisms into cultivation systems has emerged as a promising strategy to improve soil quality, enhance crop resilience, and support more sustainable agricultural practices (Devaux *et al*., 2021).

Among plant-associated microbes, plant growth-promoting rhizobacteria (PGPR) belonging to the genus *Pseudomonas* are considered particularly promising due to their versatile capacity to enhance nutrient acquisition, stimulate plant growth, and increase tolerance to biotic and abiotic stresses (Pieterse *et al*., 2021; Shuang *et al*., 2022; Sharma *et al*., 2025b). A growing body of research demonstrates that various *Pseudomonas* species and strains can positively influence plant productivity and health, either directly by promoting growth or indirectly by mitigating environmental stress and suppressing pathogens (Vraný and Fiker, 1984; Sturz, 1995; Arseneault *et al*., 2015; Guyer *et al*., 2015; Oni *et al*., 2015; Mousa *et al*., 2024; Burr *et al*., 2025; Khoshru *et al*., 2025; Ollio *et al*., 2025).

The effectiveness of PGPR inoculation depends on several factors, including the nature of the association between bacterial strains and the host plant, environmental conditions, and the chemical signalling that mediates plant–microbe interactions. Chemical profiling of *Pseudomonas* exudates has revealed that metabolite composition is highly strain specific and includes compounds associated with both plant growth regulation (e.g. auxins) and defence responses [e.g. hydrogen cyanide (HCN)] (Workentine *et al*., 2010).

Microbial consortia may outperform single-strain inoculants by providing complementary functions (Bradáčová *et al*., 2019; Christel *et al*., 2021). However, the mechanisms underlying such synergistic interactions remain poorly understood, particularly regarding how combined PGPR treatments influence plant developmental and defence signalling networks. Understanding how microbial consortia influence plant signalling networks is therefore a key step towards developing reliable microbial-based strategies for sustainable crop production.

In this context, we focus on two well-characterized PGPR strains, *Pseudomonas protegens* CHA0 and *P. simiae* WCS417, which differ in their dominant functional traits. While *P. protegens* is known for producing metabolites associated with growth promotion and biocontrol, *P. simiae* has been shown to strongly induce host immune signalling and systemic resistance. Their contrasting functional profiles therefore provide a suitable system for exploring whether complementary microbial activities can generate synergistic effects on plant development and defence regulation.

This question is particularly relevant for major food crops such as potato (*Solanum tuberosum*), the most important dicot crop globally and a key contributor to food and nutritional security (Burgos *et al*., 2020; Mickiewicz *et al*., 2022). Despite a recent decline in global harvested area for potato from 18.1 Mha in 2022 to 16.8 Mha in 2023 (FAOSTAT, 2022), productivity gains reflect advances in crop management and agricultural innovation that allow higher yields and more efficient resource use. At the same time, climate change, soil degradation, and yield instability challenge the chemical-intensive legacy of industrial agriculture. Against this background, the plant-associated microbiome is increasingly recognized as a promising yet underutilized resource for improving crop productivity and resilience (Koch *et al*., 2020; Christel *et al*., 2021; Devaux *et al*., 2021).

Northern Scandinavia represents an emerging region for climate-resilient seed potato production, where sustainable soil management approaches such as PGPR-based strategies may contribute to improved productivity and stability (Albrectsen *et al*., 2025). Understanding how beneficial microbial interactions influence potato growth and defence under controlled conditions is therefore an important step towards developing microbial solutions that may later be evaluated in field systems.

Understanding how microbial inoculants influence plant developmental signalling requires integrating physiological responses with molecular analyses of key regulatory pathways. Potato represents a suitable model system for studying plant–microbe interactions at the molecular level, as key regulatory pathways controlling tuber development and defence signalling have been extensively characterized. Tuberization is regulated by a network of mobile signals and transcriptional regulators that integrate environmental cues such as daylength and temperature (Singh *et al*., 2023; Roeder *et al*., 2025). A central component of this network is SELF-PRUNING 6A (StSP6A), a homologue of the protein encoded by the Arabidopsis *FLOWERING LOCUS T* gene that functions as a mobile tuberigen transported from leaves to stolons where tuber formation is initiated (Navarro *et al*., 2011; Plantenga *et al*., 2019; Bao *et al*., 2025). The transcription factor StBEL5 acts as a mobile RNA signal that promotes tuberization partly by activating StSP6A expression (Cho *et al*., 2015; Sharma *et al*., 2016), while StSP5G represses StSP6A under non-inductive long-day conditions through the CONSTANS-LIKE signalling pathway (Abelenda *et al*., 2016; Kondhare *et al*., 2021; Guillemette *et al*., 2024). Together these regulators integrate environmental signals to coordinate the timing of tuber initiation.

Emerging evidence suggests that plant defence signalling networks also contribute to growth stimulation by PGPR. For example, *P. protegens* and *P. simiae* can modulate jasmonic acid (JA)-related genes including those encoding allene oxide cyclase (AOC) and the MYC2 transcription factor, which play important roles in both defence priming and developmental transitions associated with tuberization (Kondhare *et al*., 2020). In addition, PGPR can activate salicylic acid- (SA) and ethylene-responsive pathways, including genes such as Callose Synthase 12 (*CalS12*) and Ethylene Response 1 (*ETR1*), enhancing structural defence responses and contributing to systemic acquired resistance (Pieterse *et al*., 2014; Gao *et al*., 2015).

These multilayered signalling responses suggest that beneficial microbes may influence plant performance by modulating both developmental and immune regulatory networks. Understanding this regulatory interplay is central to harnessing PGPR for crop improvement. However, empirical studies linking microbial presence to the regulation of core developmental signalling pathways in crop plants remain limited.

In this study, we hypothesized that co-inoculation with *P. protegens* CHA0 and *P. simiae* WCS417 would exert synergistic effects on potato growth and defence regulation. We further propose that these effects arise from complementary microbial functions influencing nutrient acquisition, hormone signalling, and immune priming.

These combined bacterial effects would be reflected in coordinated changes in plant developmental and defence signalling networks, ultimately influencing tuber formation and plant performance. To test this hypothesis, we investigated the effects of bacterial exudates from these two strains on two potato cultivars (cv. Désirée and cv. Mandel). Specifically, we examined (i) the metabolite profiles of bacterial exudates produced by the two PGPR strains individually and in combination, (ii) the effects on potato growth and development of single versus combined inoculation to determine whether bacterial interactions produce additive or synergistic responses, (iii) the regulation of growth- and defence-related signalling pathways, and (iv) spatiotemporal gene expression dynamics across tissues and developmental stages.

To our knowledge, this study provides one of the first integrated analyses combining metabolomic profiling of PGPR exudates with analyses of potato developmental responses and gene expression dynamics across multiple tissues and developmental stages. To address these questions, we combined metabolomic analyses of bacterial exudates with physiological measurements of plant growth and tuber development, together with spatiotemporal analyses of defence- and development-related gene expression in potato plants exposed to single or combined bacterial treatments.

## Materials and methods

### Bacterial cultures

In this study, two strains of *Pseudomonas* were used: *Pseudomonas protegens* CHA0 (Voisard *et al*., 1989; NCBI: txid1124983) and *Pseudomonas simiae* WCS417 (Pieterse *et al*., 2021), hereafter mostly referred to as PP and PS. Green fluorescent protein (GFP)-tagged derivatives of both strains were also included (*P. protegens* CHA0-GFP2; Péchy-Tarr *et al*., 2013, and *P. simiae* WCS417-GFP). Cultures were maintained on King’s B (KB) agar plates and stored at −80 °C in 20% (v/v) glycerol stocks.

### Characterization of bacterial growth-promoting traits

Plant growth-promoting traits of the two bacterial cultures, PP and PS, were assessed under laboratory conditions at the Umeå Plant Science Centre, Sweden. The assays described below included measurements of indole-3-acetic acid (IAA) production, associated with root development and plant growth; phosphate solubilization, enhancing phosphorus (PO_4_^−^) availability; and HCN production, linked to antagonistic activity against plant pathogens.

In addition, bacterial colonization and localization were examined using confocal microscopy. Metabolic profiles of bacterial exudates were analysed using LC-MS for cultures grown individually and in combination.

#### Indole-3-acetic acid production

IAA production was determined colorimetrically using Salkowski reagent following the method of Batista *et al*. (2020). Bacterial strains were inoculated into Luria–Bertani (LB) broth supplemented with 0.5 mg ml^−1^  L-tryptophan and incubated at 28 °C for 10 d with shaking at 120 rpm. Samples (1 ml) were collected after 48, 72, 96, 120, and 240 h, and filtered through Munktell filter paper (110 mm diameter).

An equal volume (1 ml) of Salkowski reagent was added to each sample, followed by incubation in the dark at room temperature for 30 min. Uninoculated LB broth supplemented with L-tryptophan and incubated under identical conditions served as a negative control. Absorbance was measured at 535 nm using a UV–Vis spectrophotometer (Hermle Labortechnik GmbH, Wehingen, Germany).

#### Phosphate solubilization assay

Phosphate solubilization ability was assessed using Pikovskaya agar following the method described by Batista *et al*. (2020). Each bacterial strain was spot-inoculated at the centre of the agar plate using 5 µl of an overnight culture (OD_600_ ∼1.0), and plates were incubated at 28 °C for 7 d.

Phosphate solubilization was indicated by the formation of a clear halo surrounding the bacterial colony. The diameters of the colony and the halo zone were measured using a digital caliper. The phosphate solubilization index (PSI) was calculated as:


(1 ) $$\mathrm{PSI}=\frac{\mathrm{colony}\;\mathrm{diameter}+\mathrm{halo}\;\mathrm{zone}\;\mathrm{diameter}}{\mathrm{colony}\;\mathrm{diameter}}.$$

#### Hydrogen cyanide production

HCN production by bacterial isolates was assessed following the protocol of Lotfi *et al*. (2022). Plates were streaked with each bacterial strain, and a piece of filter paper (Munktell filter paper) soaked in a solution containing 2% (w/v) sodium carbonate and 0.5% (w/v) picric acid was affixed to the inner surface of the Petri dish lid. The plates were sealed with Parafilm to retain volatile compounds and incubated at 28 °C for 5 d in the dark. Uninoculated plates served as negative controls. HCN production was indicated by a colour change of the filter paper from deep yellow to orange or reddish-brown.

#### Confocal microscopy for validation of bacterial colonization

To assess the ability of PGPR strains to colonize potato roots and visualize their spatial distribution within root tissues, confocal laser scanning microscopy (CLSM) was performed using fluorescently tagged bacterial strains. CHA0-GFP and WCS417-GFP were grown overnight at 28 °C in KB medium supplemented with gentamicin (20 µg ml^−1^) and tetracycline (15 µg ml^−1^), respectively. Bacterial cultures were washed three times with 10 mM magnesium sulfate (MgSO_4_) and adjusted to OD_600_ = 0.5. Roots of 1-month-old *S. tuberosum* cv. Désirée plantlets grown on Murashige and Skoog (MS-10) medium (4.4 g l^−1^ MS salts, 10 g l^−1^ sucrose, 3 g l^−1^ Gelrite, and 8 g l^−1^ agar; pH 5.8 ± 0.1) (Murashige and Skoog, 1962) were inoculated with 200 µl of the bacterial suspension (either CHA0-GFP or WCS417-GFP).

Confocal microscopy was performed using a Zeiss LSM 980 system (Carl Zeiss AG, Germany) equipped with a Plan-Apochromat ×40/1.2 NA objective. Fluorescent imaging was performed using GFP-tagged bacterial strains, allowing visualization of bacteria in the green channel (Bloemberg *et al*., 1997). After 24 h, 1 cm root tip segments were excised from control and treated plantlets and counterstained with propidium iodide (PI; 100 µg ml^−1^ in 10 mM MgSO_4_) for 2 min to visualize plant cell walls (red fluorescence). Samples were mounted on microscope slides with coverslips and imaged immediately. GFP fluorescence was excited at 488 nm and PI at 561 nm, with emission detected using BP 525/50 and BP 629/62 filters, respectively. Images were acquired and processed using ZEN Blue 3.8 (Carl Zeiss AG, Germany). Bacterial colonization was assessed qualitatively based on visual inspection of fluorescence signals. Root colonization assays were conducted using cv. Désirée plantlets due to their robust *in vitro* root development.

### Metabolomic profiling

Metabolomic analyses were performed to characterise bacterial metabolic profiles when the strains were grown individually or in combination. LC-MS analysis was carried out at the Swedish Metabolomics Center (Umeå, Sweden).

PP and PS were cultured separately or together (PPPS) for 72 h at 28 °C in KB medium. Each treatment was prepared in three biological replicates (nine cultures in total). For each sample, 1 ml of culture (OD_600_ = 1.0) was centrifuged at 3500 *g* for 5 min. Supernatant and pellet fractions were collected and processed separately, yielding two sample types. Samples 1–3 corresponded to PP supernatant, 4–6 to PS supernatant, and 7–9 to PPPS supernatant, whereas samples 10–12 represented PP pellet, 13–15 PS pellet, and 16–18 PPPS pellet.

Metabolite extraction was performed according to Jiye *et al*. (2005). Briefly, 400 µl of extraction buffer (80:20 v/v methanol:water) containing internal standards was added to each sample. Samples were homogenized with a tungsten bead in a mixer mill at 30 Hz for 3 min, after which the bead was removed and the samples were centrifuged at 4 °C and 18 620 *g* for 10 min. A 200 µl aliquot of supernatant was transferred to microvials for LC-MS analysis and evaporated to dryness using a speed-vac concentrator. Samples were stored at −80 °C until analysis. Prior to LC-MS analysis, dried extracts were reconstituted in 10 µl of methanol and 10 µl of water. Samples were analysed in a randomized run order. Each sample was first analysed in positive ionization mode, after which the instrument was switched to negative mode and a second injection was performed.

Chromatographic separation was performed on an Agilent 1290 Infinity UHPLC system (Agilent Technologies, Waldbronn, Germany). A 2 μl aliquot of each sample was injected onto an Acquity UPLC HSS T3 C18 column (2.1×50 mm, 1.8 µm) coupled to a VanGuard pre-column (2.1×5 mm, 1.8 µm; Waters Corporation, Milford, MA, USA) maintained at 40 °C. The mobile phases consisted of (A) water containing 0.1% formic acid and (B) 75:25 acetonitrile:2-propanol containing 0.1% formic acid, with a flow rate of 0.5 ml min^−1^. The linear gradient started at 0.1% B and increased to 10% B over 2 min, followed by an increase to 99% B over 5 min and a hold at 99% B for 2 min. The gradient was then returned to 0.1% B for 0.3 min, after which the flow rate was increased to 0.8 ml min^−1^ for 0.5 min and held for 0.9 min before returning to 0.5 ml min^−1^ for the next injection.

MS detection was performed using an Agilent 6546 Q-TOF mass spectrometer equipped with a Jet Stream electrospray ion source operating in positive or negative ionization mode. Instrument parameters were identical for both modes except for the capillary voltage. A reference interface was used for accurate mass calibration. The reference ions purine (4 µM) and HP-0921 [Hexakis (1H,1H,3H-tetrafluoropropoxy)phosphazine](1 µM) were continuously infused at 0.05 ml min^−1^. Monitored ions were *m/z* 121.0509 and 119.03632 for purine, and *m/z* 922.0098 and 966.000725 for HP-0921 in positive and negative mode, respectively.

The gas temperature was set to 150 °C, drying gas flow to 8 l min^−1^, and nebulizer pressure to 35 psig. Sheath gas temperature was 350 °C with a flow rate of 11 l min^−1^. Capillary voltage was set to 4000 V in both ionization modes. The nozzle voltage was 300 V, fragmentor voltage 120 V, skimmer voltage 65 V, and OCT 1 RF Vpp 750 V. Collision energy was set to 0 V. Mass spectra were acquired over an *m/z* range of 70–1700 in centroid mode at an acquisition rate of 4 scans s^−1^.

Data processing was performed using Agilent MassHunter Profinder v. B.10.0.2 (Agilent Technologies, Santa Clara, CA, USA). Features were matched against an in-house LC-MS library generated from authentic standards analysed under identical chromatographic and mass spectrometric conditions using the Batch Targeted Feature Extraction workflow with an *m/z* tolerance of 20 ppm and a retention time window of 0.1 min.

Metabolite annotation confidence was assigned according to established metabolomics reporting standards (Sumner *et al*., 2007; Schymanski *et al*., 2014; Schrimpe-Rutledge *et al*., 2016). Level 1 represented confirmed identification using authentic standards with agreement in *m/z*, retention time, and MS/MS fragmentation. Level 1* indicated confirmed isomeric mixtures that could not be chromatographically resolved. Level 2 corresponded to putative structural annotation based on spectral library matches or diagnostic MS/MS evidence. Level 3 indicated tentative annotation or compound class assignment based on accurate mass and database searches. Level 4 corresponded to features with assigned molecular formula only, and Level 5 represented unique features with measured accurate mass but no structural assignment. Metabolite abundance was estimated from peak areas. Features not meeting annotation confidence criteria were considered below the detection limit and treated as missing values in subsequent analyses.

### Plant material

Two potato cultivars (*S. tuberosum* L.) were used in this study. The cv. Mandel was selected for its regional significance as a landrace commonly cultivated in northern Sweden (Veteläinen *et al*., 2005), whereas cv. Désirée is a widely adapted cultivar known for its agronomic stability across diverse environments. Tubers of cv. Mandel were obtained from the SLU field station at Röbäcksdalen (Västerbotten, Sweden), while cv. Désirée tubers were obtained from the breeding company Danespo A/S, Give, Denmark. Tubers were stored at 5 °C until sprouting.

Sprouts were excised with a scalpel and surface-sterilized in 1% sodium hypochlorite (NaOCl) for 20 min, followed by three rinses in sterile distilled water, according to the protocol of Kieu *et al*. (2021). Sprouts were cut into 1 cm explants and cultured *in vitro*. Meristem-derived plantlets were propagated in glass culture tubes containing modified MS-10 medium. Cultures were maintained at 18–22 °C under a 16 h light/8 h dark photoperiod with a light intensity of 100 µmol m^−2^ s^−1^. Contaminated explants were discarded during subculturing until only contamination-free plantlets remained in culture.

### Inoculation with *P. protegens* and *P. simiae*

After 4 weeks of *in vitro* cultivation, 40 plantlets of each cultivar were transplanted into 1 litre pots containing an autoclaved sandy soil–vermiculite mixture (3:1, v/v), with one plantlet per pot. For each cultivar, 10 plants served as uninoculated controls, while the remaining 30 plants were assigned to three bacterial treatments (10 replicates per treatment): inoculation with PP, PS, or a combination of both strains (PPPS).

Plants were grown in a greenhouse under controlled conditions with a 16 h light/8 h dark photoperiod at ∼20 °C, a light intensity of 200 µmol m^−2^ s^−1^, and 60% relative humidity.

For bacterial inoculation, *P. protegens* CHA0 and *P. simiae* WCS417 were grown overnight (16 h) at 28 °C in KB medium with shaking. Cultures were centrifuged at 3500 *g* for 5 min, washed three times by resuspending the pellet in 10 mM MgSO_4_, and adjusted to OD_600_=0.6, corresponding to ∼3–5×10^8^ colony-forming units (CFU) ml^−1^. For the dual-inoculation treatment, equal volumes (1:1) of each bacterial suspension with the same OD_600_ were mixed prior to application.

Each bacterial treatment was applied to 10 plants per cultivar by pipetting 2 ml of bacterial suspension (∼6–10 × 10^8^ CFU) into the soil 2–3 cm from the plant base at a depth of ∼5 cm. Control plants received 2 ml of sterile 10 mM MgSO_4_ using the same procedure.

The experiment followed a randomized design, with plants distributed across four greenhouse tables and repositioned weekly. Pots were irrigated with sterile distilled water and half-strength Hoagland’s No. 2 basal salt solution (Sigma-Aldrich).

Following bacterial inoculation, two plants per treatment and cultivar were harvested at bi-weekly intervals. Soil was gently removed from the roots, and root and leaf tissues were immediately flash-frozen in liquid nitrogen and stored at −80 °C. No tubers had developed after 2 weeks; however, mini-tubers formed at later harvests were collected and stored at 4 °C.

After 8 weeks, above-ground plant parts from the remaining plants were harvested, while the below-ground parts were left in the soil for an additional 4 weeks to allow tuber periderm maturation. Mature mini-tubers were then harvested and stored at 4 °C for tuber quality analyses.

### Plant phenotyping

Plant height was measured in centimetres as the distance from the soil surface to the apex of the tallest stem of each plant. Measurements were performed 2, 4, and 6 weeks after bacterial inoculation using ten, eight, and six biological replicates per treatment and cultivar, respectively.

Chlorophyll measurements were performed at the same time points as plant height measurements. They were assessed non-destructively using a Dualex 4 Scientific sensor (Dx4; FORCE-A, Paris, France). Measurements were taken from three fully expanded young leaves randomly selected from the upper canopy of each plant. The mean of the three readings was used to calculate a single chlorophyll index for each replicate.

### Tuber quality analyses

Tuber quality was evaluated by measuring starch and soluble sugar content, specific gravity, and ascorbic acid concentration.

#### Starch and soluble sugar quantification

Starch and soluble sugars were quantified using the Anthrone method (Hansen and Møller, 1975; Sadasivam, 1996) with six biological replicates (mini-tubers) per treatment (*n* = 6). Fresh tuber tissue (1 g) was homogenized in 15 ml of 80% ethanol and centrifuged at 11 180 *g* for 10 min. Analyses were performed in triplicate. The resulting pellet was used for starch extraction, while the supernatant was used for soluble sugar analysis.

For starch quantification, a mixture of 52% perchloric acid (6.5 ml) and water (5 ml) was added to the pellet and incubated at 2 °C for 20 min. After centrifugation at 11 180 *g* for 15 min, the supernatant was collected. This extraction step was repeated three times, and the supernatants were pooled and diluted (1:5). The diluted extract was reacted with Anthrone reagent in a boiling water bath for 8 min. After cooling to room temperature, absorbance was measured at 630 nm using a UV–Vis spectrophotometer (Hermle Labortechnik GmbH, Wehingen, Germany). Glucose concentrations were determined from a standard curve (20–100 µg ml^−1^), and starch content was calculated by multiplying glucose concentration by 0.9 (Sadasivam, 1996).

For soluble sugar analysis, the ethanol supernatant was incubated at 0 °C for 30 min and then mixed with ice-cold Anthrone reagent (1:5, v/v). The mixture was incubated at 85 °C for 10 min and subsequently cooled in an ice bath. Absorbance was measured at 630 nm, and sugar concentrations were determined using the same glucose standard curve.

#### Specific gravity

Specific gravity was determined for six tubers per treatment (*n* = 6). Tubers were washed, dried, and weighed in air, followed by submerged weighing in water using a mesh net. Specific gravity was calculated according to Müller *et al*. (2023):


(2 ) $$\mathrm{Specific}\;\mathrm{gravity}\;=\;\frac{\mathrm{Weight}\;\mathrm{in}\;\mathrm{air}}{\mathrm{Weight}\;\mathrm{in}\;\mathrm{air}-\mathrm{Weight}\;\mathrm{in}\;\mathrm{water}}.$$

#### Ascorbic acid content

Ascorbic acid content was determined following the method described by Ranganna (1986). Fresh tuber tissue (5 g) was homogenized in 5 ml of 1.0% hydrochloric acid (HCl) and centrifuged at 11 180 *g* for 10 min. Absorbance of the supernatant was measured at 243 nm using the same UV–Vis spectrophotometer described above. Ascorbic acid concentrations were calculated from a standard curve and expressed as µg g^−1^ FW (*n* = 6 per treatment).

### Gene expression analyses

Gene expression analyses were conducted to examine tuberization-related genes and hormonal stress signalling markers across leaf, root, and tuber tissues collected from *S. tuberosum* cv. Désirée plants at 2, 4, and 6 weeks post-inoculation.

Total RNA was extracted from frozen tissue samples using the RNAqueous™ Kit (Invitrogen, Carlsbad, CA, USA) according to the manufacturer’s instructions. Leaf, root, and tuber samples were flash-frozen in liquid nitrogen and stored at −80 °C prior to extraction. RNA purity and concentration were assessed using a NanoDrop ND-1000 spectrophotometer (Saveen Werner Life Science, Sweden).

For each sample, 50 ng of RNA was used in one-step quantitative reverse transcription–PCRs (qRT–PCRs) with the QuantiNova® SYBR Green RT–PCR Kit (Qiagen, Valencia, CA, USA), which includes integrated genomic DNA removal and reverse transcription. qRT–PCR was performed using a CFX96 Real-Time PCR Detection System (Bio-Rad Laboratories, Hercules, CA, USA).

Primers were designed using Primer3 software and are listed in Supplementary Dataset S1. Each reaction was performed in technical triplicate using RNA extracted from independent biological samples. The elongation factor 1-alpha (*EF1α*) gene was used as a reference for normalization (Elkobrosy *et al*., 2022), supported by studies demonstrating stable expression in potato (Nicot *et al*., 2005). Relative expression patterns were consistent across biological replicates and treatment comparisons, indicating stable normalization across the experimental conditions.

Thermal cycling conditions consisted of reverse transcription at 45 °C for 10 min, initial denaturation at 95 °C for 5 min, followed by 40 cycles of 95 °C for 5 s, 60 °C for 30 s, and 72 °C for 1 min. Relative expression levels were calculated using the 2^−ΔΔ^CT method (Livak and Schmittgen, 2001), comparing each treatment with the control within the same tissue and time point.

### Classification of expression patterns and statistical analysis

For each gene × tissue × time point combination, fold changes relative to the control were calculated for the PS, PP, and PPPS treatments. Fold change values were derived from group-wise means [e.g. Δ_PS = mean(PS)/mean(Control)]. Statistical significance of treatment effects relative to the control was assessed using unpaired two-tailed *t*-tests (α=0.05). Based on these values, expression responses under the combined treatment (PPPS) were classified into the following categories: (i) synergistic—PPPS fold change significantly exceeded or fell below the mean of PS and PP by >20%; (ii) additive—PPPS fold change was within ±5% of the mean of PS and PP; (iii) dominant—PPPS matched either PS or PP (±5%), while PS and PP differed from each other (>5%); (iv) neutral—none of the treatments differed significantly from the control; and (v) other–patterns not fitting the categories above

These categories were used to identify potential interaction effects between the two bacterial strains on gene expression. Summary tables and heatmaps were generated to visualize tissue-specific expression patterns and interaction types.

### Statistical analyses

Statistical analyses were performed using R software (R Core Team, 2024).

Data normality for plant growth, tuber quality parameters, and bacterial growth-promoting traits was assessed using the Shapiro–Wilk test. When data were not normally distributed, the Kruskal–Wallis test was applied to compare treatment effects.

Two-way ANOVA was used to evaluate the effects of treatments and incubation time on IAA production. For PSI values, independent-samples *t*-tests were used to compare treatment groups assuming equal variances.

For normally distributed datasets, ANOVA was performed to evaluate the effects of treatment, cultivar, experimental time, and their interactions on measured parameters. For factorial designs involving non-normally distributed data, the aligned rank transform (ART) method was applied using the ARTool package to enable ANOVA on aligned ranks.

Principal component analysis (PCA) was conducted on metabolomics data using the rda() function in the vegan package in R.

## Results

### Plant growth-promoting traits of *P. protegens* CHA0 and *P. simiae* WCS417

**Fig. 1.**
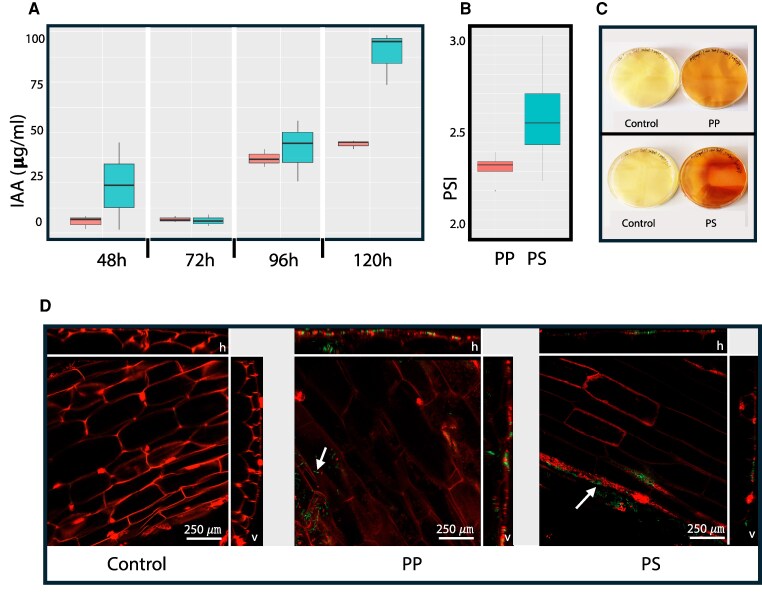
Characterization of plant growth-promoting traits in the bacterial strains used in this study: *Pseudomonas protegens* CHA0 (PP, red) and *P. simiae* WCS417 (PS, blue). (A) Boxplot showing indole acetic acid (IAA) production at 48, 72, 96, and 120 h post-inoculation, measured colorimetrically using Salkowski reagent (*n* = 3). (B) Boxplot showing the phosphate solubilization index (PSI), PO_4_^–^ determined after 7 d via spot inoculation on tricalcium phosphate agar plates (*n*=3). (C) Hydrogen cyanide (HCN) production compared with sterile water controls, tested on King’s B agar medium supplemented with 0.44% (w/v) glycine. (D) Confocal microscopy images showing bacterial localization (GFP = green fluorescent protein, green) in association with potato root cells 24 h post-inoculation. Control roots were treated with sterile water (cell walls counterstained with propidium iodide, red). PP-treated roots were inoculated with GFP-tagged *P. protegens* CHA0, while PS-treated roots were inoculated with GFP-tagged *P. simiae* WCS417. Orthogonal views are provided, with horizontal (h) and vertical (v) panels representing optical sections along the *x–z* and *y–z* axes. Arrowheads indicate regions of bacterial colonization. Images were acquired using a confocal microscope equipped with a Plan-Apochromat ×40/1.2 NA water immersion objective. Scale bar = 250 µm.

Plant growth-promoting traits of PP and PS were characterized *in vitro* (Fig. 1A–D). IAA production increased over time in both strains, with measurements taken at 48, 72, 96, and 120 h post-inoculation. At 120 h, PS produced significantly higher levels of IAA than PP (Fig. 1A).

Both strains were able to solubilize inorganic phosphate, as indicated by their PSI values. PSI was significantly higher in PS (∼2.7) than in PP (∼2.1) (Fig. 1B).

Qualitative assays further showed that both strains produced HCN, with PS exhibiting a stronger colour response than PP (Fig. 1C).

### Colonization of *P. protegens* CHA0 and *P. simiae* WCS417 in the potato root

CLSM revealed colonization of potato roots by GFP-tagged *P. simiae* WCS417 and *P. protegens* CHA0. No GFP fluorescence was detected in root tissues of uninoculated control plants (Fig. 1D).

In inoculated plants, GFP-expressing bacterial cells were observed on the root surface and within intercellular spaces of root tissues within 24 h after inoculation, indicating that both strains rapidly established associations with potato roots.

### Metabolite analyses of *P. protegens* and *P. simiae*: individually and combined

**Fig. 2.**
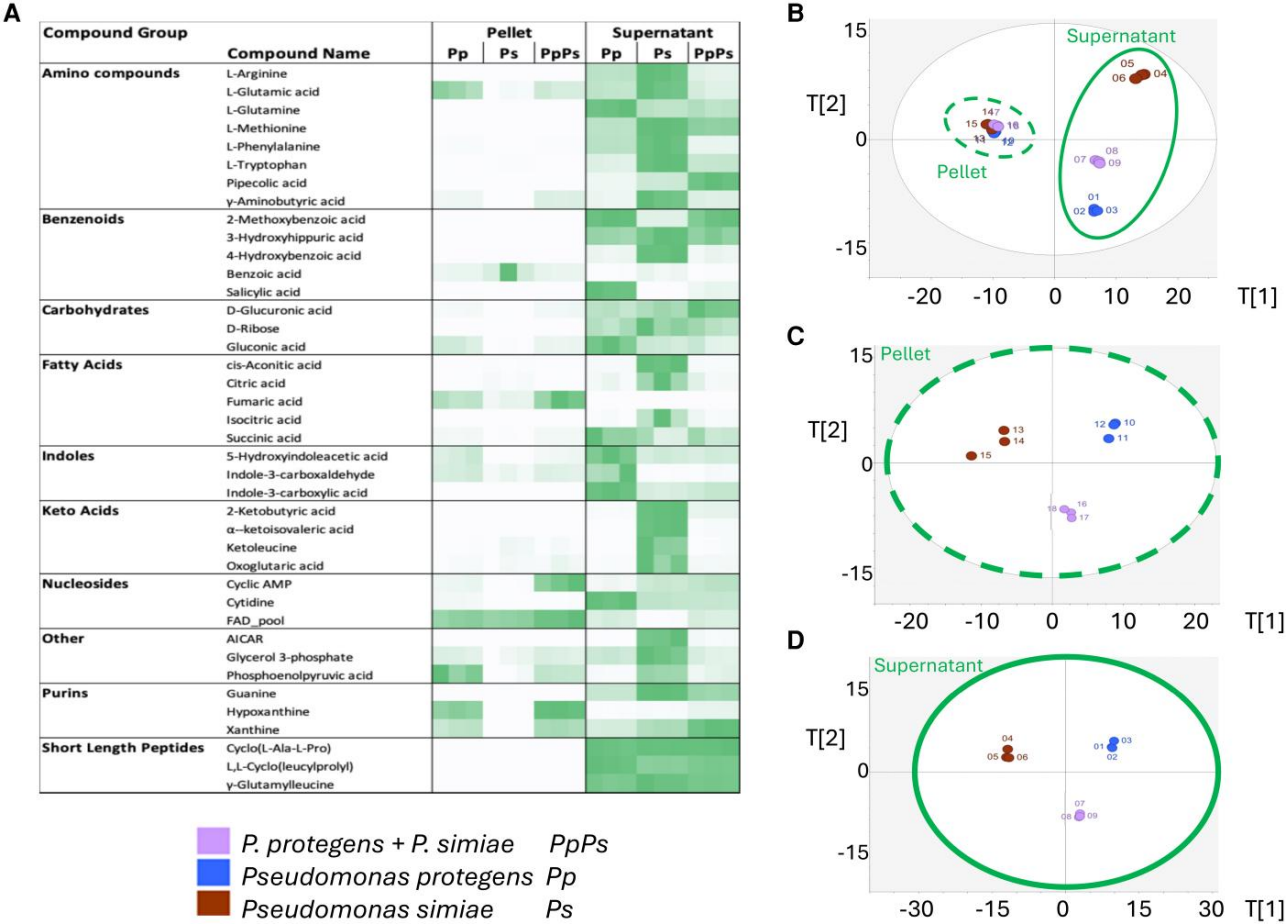
Metabolomic profiling of pellet and supernatant fractions from three bacterial culture treatments: *Pseudomonas protegens* (PP), *P. simiae* (PS), and their combination (PPPS). (A) Heatmap showing selected metabolic products from a targeted list of ∼180 metabolites (see Supplementary Dataset S2). Metabolites were quantified using LC-MS in negative ionization mode, processed with internal standards, and normalized within each metabolite across samples to allow relative comparison. Metabolites are grouped by chemical class, and relative abundances are visualized in green colour shading. White cells indicate values below the LC-MS detection limit. Samples were analysed in triplicate for both insoluble (pellet) and soluble (supernatant) fractions (*n*=18). Pellet samples: PP (10–12), PS (13–15), PPPS (16–18); corresponding supernatant samples: PP (1–3), PS (4–6), PPPS (7–9). (B) Principal component analysis (PCA) of all samples. (C) PCA based on metabolites detected in pellet fractions only. (D) PCA based on metabolites from supernatant fractions only.

LC-MS was used to analyse metabolites present in pellet and supernatant fractions of PP, PS, and their combined culture (PPPS). Samples were analysed in triplicate for each treatment (OD_600_=1.0), resulting in a total of 18 samples (3 treatments × 3 replicates × 2 fractions). In total, 181 metabolites were annotated across all samples (selected metabolites in Fig. 2A; all metabolites in Supplementary Dataset S2).

PCA based on all annotated metabolites revealed clear separation between pellet and supernatant fractions, with extracellular metabolites accounting for the largest variation among samples (Fig. 2B). When analysed separately, both pellet and supernatant datasets also showed distinct clustering according to bacterial treatment (Fig. 2C, D), indicating treatment-specific metabolic profiles.

Annotated metabolites were assigned to several compound classes, including amino acids, phenolics, tricarboxylic acid (TCA) cycle intermediates, fatty acids, nucleotides, carboxylic acids, sugars, and auxin-related compounds (Fig. 2A).

Overall, many metabolites were more abundant in supernatant fractions than in pellets. Amino acids were particularly abundant in the supernatant of PS cultures, although in some cases the combined culture (PPPS) exhibited higher levels than either single strain. Several amino acids, including L-glutamic acid, L-glutamine, and L-arginine, were detected across treatments.

Benzenoid compounds, including SA derivatives, were predominantly detected in supernatant fractions and varied among bacterial treatments. Carbohydrate-related metabolites, such as glucuronic acid, were also enriched in supernatant samples.

In contrast, fatty acids were detected at comparable or higher levels in pellet fractions than in supernatants. This distribution suggests differential intracellular and extracellular metabolite allocation among the bacterial treatments.

### Dual *Pseudomonas* strains alter central carbon metabolism

Metabolite profiling of the extracellular medium revealed distinct differences between single-strain (PP and PS) and dual-strain (PPPS) cultures. The metabolic composition of the single-strain supernatants differed substantially. The PS supernatant was enriched in indoles and their derivatives, whereas the PP culture contained higher levels of amino acids and several signalling-related metabolites, including γ-aminobutyric acid (GABA), α-ketoisovaleric acid, oxoglutaric acid (α-ketoglutarate), and hippuric acid.

In the dual-strain treatment (PPPS), several metabolites exhibited non-additive accumulation patterns compared with the single-strain cultures. Notably, the dual culture showed elevated levels of metabolites such as xanthine, pipecolic acid, L-methionine, cAMP, and FAD.

In contrast, multiple intermediates of the TCA cycle (including citric acid, isocitric acid, α-ketoglutaric acid, succinic acid, and fumaric acid) were reduced in the PPPS treatment relative to either single strain. Malic acid was moderately elevated, whereas phosphoenolpyruvate (PEP) and GABA showed intermediate reductions.

Four metabolites were reduced to <10% of the levels detected in the single-strain cultures: AICAR (5-aminoimidazole-4-carboxamide ribonucleotide), ketoleucine, α-ketoisovaleric acid, and *cis*-aconitic acid [fold change < 0.1; calculated as PPPS/mean(PP, PS)].

### Effects on root and stolon development

**Fig. 3.**
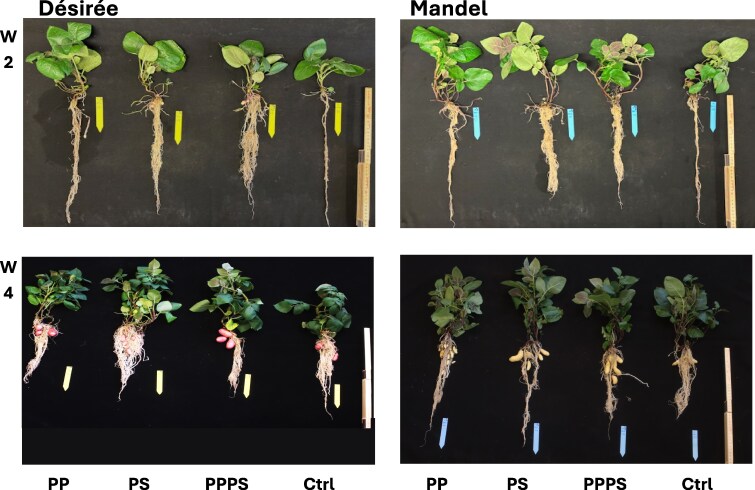
Root architecture phenotyping of potato plants following bacterial treatments. Representative images of potato cultivars Désirée (left) and Mandel (right), taken at 2 weeks (top) and 4 weeks (bottom) post-inoculation with plant growth-promoting rhizobacteria (PGPRs). For each cultivar and time point, plants are arranged from left to right according to treatment: PP (*P. protegens* CHA0), PS (*P. simiae* WCS417), PPPS (combined inoculation with PP and PS), and C (control, inoculated with sterile water).

In both cv. Désirée and cv. Mandel, inoculation with PP, PS, or PPPS enhanced root development compared with the uninoculated control (Fig. 3). Two weeks after inoculation, treated plants exhibited increased lateral root proliferation and early stolon formation, a developmental stage that was not observed in control plants at this time point.

By 4 weeks, all inoculated plants displayed more extensive and highly branched root systems than untreated controls. The magnitude of the response differed between cultivars, with cv. Désirée showing a stronger response to bacterial inoculation than cv. Mandel.

### Effects on chlorophyll content, plant height, and tuber quality

**Fig. 4.**
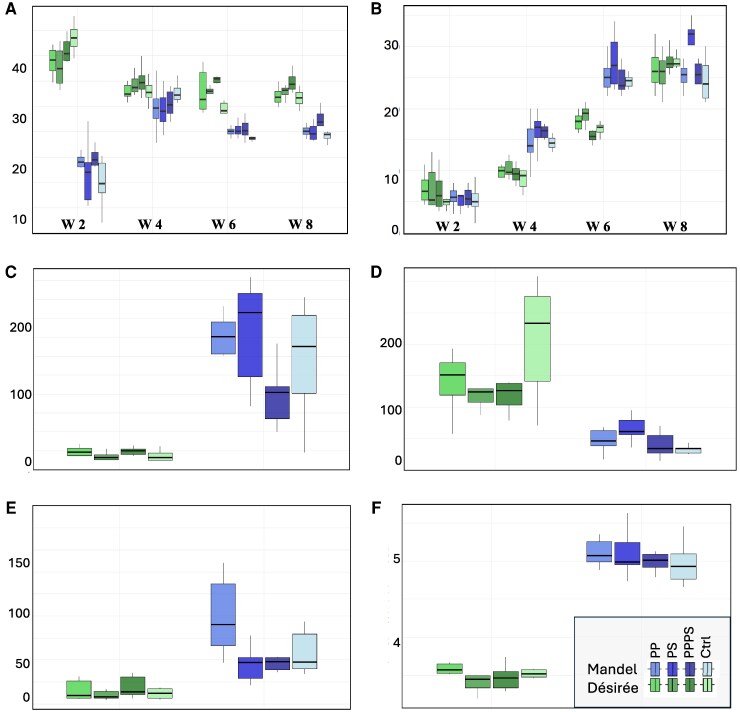
Effects of rhizobacterial treatments on above-ground growth and tuber quality traits in potato (*Solanum tuberosum* L.). Boxplots show the effects of individual and combined bacterial inoculations on six phenotypic traits measured in two cultivars: cv. Désirée (green) and cv. Mandel (purple). Treatments included *Pseudomonas protegens* CHA0 (PP), *Pseudomonas simiae* WCS417 (PS), their combined treatment (PPPS), and a sterile water control (C), as indicated in the key. Above-ground traits were measured non-destructively at 2 week intervals up to 8 weeks post-inoculation. (A) Chlorophyll content (index); (B) plant height (cm). Tuber quality traits were assessed at harvest (12 weeks post-inoculation) and included: (C) starch content; (D) sugar content; (E) specific gravity; and (F) ascorbic acid content. Traits in (C), (D), and (F) are expressed as μg g^−1^ tuber, whereas specific gravity is dimensionless.

Chlorophyll content measured across four time points (Fig. 4A) was consistently higher in cv. Désirée than in cv. Mandel. Statistical analysis revealed significant effects of cultivar (*P* < 0.001), bacterial treatment (*P* < 0.01), and time (*P* < 0.001; Table 1). A significant cultivar × treatment interaction (*P* < 0.001) indicated that the response to bacterial inoculation differed between the two cultivars.

**Table 1.** Summary of ANOVA results (aligned rank transform) assessing the effects of potato cultivar, bacterial treatment, time post-inoculation, and their interactions on plant height and chlorophyll content

			Chlorophyll		Plant height	
	df	df_res_	*F*-value	*P*-value	*F*-value	*P*-value
Cultivar (cv)	1	192	490.54	<2.2e-16***	96.46	<2.2e-16***
Treatment	3	192	6.76	0.0002***	5.09	0.002**
Time post-inoculation (tpi)	3	192	20.26	1.9e-11***	547.23	<2.2e-16***
cv×tpi	3	192	1.21*	0.31*	1.45	0.23*
cv×Treatment	3	192	182.98	<2.2e-16***	62.32	<2.2e-16***
Treatment×tpi	9	192	1.90	0.054*	1.36	0.21
cv×Treatmentt×tpi	9	192	3.35	0.0008***	1.15	0.30*

Significance levels: ***P < 0.001, **P < 0.01, *P < 0.1.

Plant height increased steadily throughout the experimental period (Fig. 4B). Growth rates differed significantly among cultivars, treatments, and sampling times (*P* < 0.001), with a significant cultivar × treatment interaction (*P* < 0.001), indicating that plant growth responses depended on both bacterial treatment and cultivar (Table 1).

Tuber quality analyses were performed on mini-tubers harvested 12 weeks after inoculation. Measurements included starch and soluble sugar contents, specific gravity, and ascorbic acid concentration (Fig. 4C–F; Table 2). No strong treatment effects were detected for these parameters. Although the combined bacterial treatment occasionally showed lower starch and sugar values, variability among replicates limited statistical significance.

**Table 2.** Summary of ANOVA results (aligned rank transform) assessing the effects of potato cultivar, bacterial treatment, and their interactions on the mini-tuber quality traits

Trait	Cultivar			Treatment			Cultivar×Treatment		
	df, df_res_	*F*	Pr(>*F*)	df, df_res_	*F*	Pr(>*F*)	df, df_res_	*F*	Pr(>*F*)
**Starch**	1, 40	100.4	1.8e-12***	3, 40	4.6	0.008**	3, 40	4.51	0.008**
**Sugar**	1, 40	59.3	2.0e-09***	3, 40	2.2	0.10	3, 40	4.40	0.009**
**SG**	1, 40	18.8	9.3e-05***	3, 40	2.3	0.09	3, 40	1.85	0.15
**AsA**	1, 40	121.3	1.1e-13***	1, 40	0. 7	0.57	1, 40	0.52	0.67

Degrees of freedom (df), *F*-values, and associated *P*-values are reported. Significance levels are indicated as: ***P < 0.001, **P < 0.01.

SG, specific gravity; AsA, and ascorbic acid. All values are µg g^–1^ exept for SG, which is unitless.

Using non-parametric ART analyses, cultivar effects were detected for all traits (Table 2). Treatment effects were observed for starch content (*P*=0.008), and significant treatment × cultivar interactions were detected for both starch and sugar contents (*P*=0.009, Table 2).

### Regulation of tuberization and defence-related gene networks

Cultivar ‘Désirée’ was selected for gene expression analyses because it showed stronger morphological responses to bacterial treatments and is widely used in functional genomics studies (Sharma *et al*., 2007). To evaluate the effects of bacterial inoculation on tuber development and defence signalling, the expression of tuberization-related genes (*StSP6A*, *StBEL5*, and *StSP5G*) and hormone-responsive genes (*MYC2*, *AOC*, *CalS12*, and *ETR1*) was analysed in leaves, roots, and tubers of *S. tuberosum* cv. Désirée. Leaf and root samples were collected at 2, 4, and 6 weeks post-inoculation, while mini-tubers were sampled at 4, 8, and 12 weeks. Complete expression data are provided in Supplementary Dataset S1.

### Expression patterns in leaves and roots

**Fig. 5.**
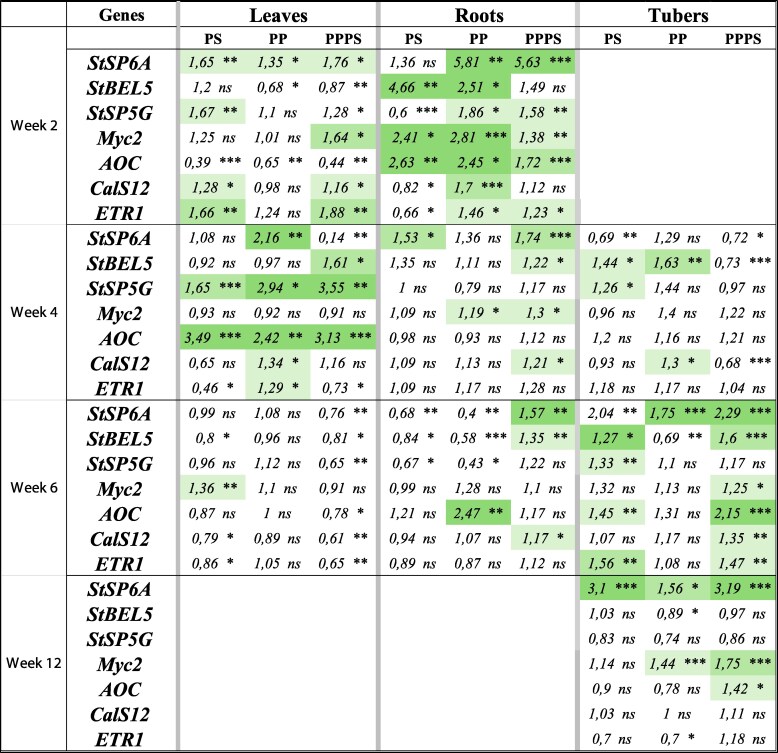
Relative expression of selected genes in leaves, roots, and tubers of potato (*Solanum tuberosum* cv. Désirée) in response to bacterial inoculation. Heatmaps show transcriptional responses to three bacterial treatments: PP (*Pseudomonas protegens* CHA0), PS (*Pseudomonas simiae* WCS417), and PPPS (combined inoculation with both strains). Tissues (leaves, roots, and tubers) were sampled at 2, 4, and 6 weeks post-inoculation. The analysed genes include: StSP6A (SELF-PRUNING 6A), StBEL5 (BEL1-like Homeobox Protein 5), StSP5G (SELF-PRUNING 5G), AOC (Allene Oxide Cyclase), CalS12 (Callose Synthase 12-like), MYC2 (Transcription Factor MYC2), and ETR1 (Ethylene Response 1). Expression values are log_2_-transformed relative to untreated controls. Up-regulation is shown in green and down-regulation in lighter shades, with colour intensity corresponding to magnitude and normalized to untreated control plants. Fold change values and significance indicators are overlaid within each heatmap cell.

Among tuberization-related genes, *StSP6A* expression remained low in leaves but was strongly induced in roots 2 weeks after inoculation, showing a 5.8-fold increase under PP treatment and a 5.6-fold increase under PPPS (Fig. 5). *StBEL5* showed negligible expression in leaves but was consistently up-regulated in roots at 2 weeks across single treatments. In contrast, *StSP5G*, a known repressor of tuberization, was up-regulated in leaves at 4 weeks across treatments but remained low in roots.

Defence-related genes displayed tissue-specific activation patterns. *AOC*, involved in JA biosynthesis, was up-regulated in roots 2 weeks after inoculation and down-regulated in leaves under both PP (2.4-fold) and PS (2.6-fold) treatments. At 4 weeks, *AOC* expression increased in leaves while remaining close to control levels in roots. By week 6, *AOC* expression declined in both tissues under PP and PPPS treatments.

The JA-responsive transcription factor gene *MYC2* showed a similar pattern, with peak expression in roots at week 2 (2.8- and 2.4-fold induction for PP and PS treatments, respectively), while remaining low in leaves. *CalS12*, associated with salicylic acid signalling, exhibited variable expression in roots following bacterial treatments and generally decreased over time. In leaves, *CalS12* was initially up-regulated under PS and PPPS treatments but declined by week 6. The ethylene-responsive gene *ETR1* showed a comparable decline in leaf expression at later time points.

### Expression patterns in tubers

Bacterial treatments generally increased gene expression levels in tubers, although *StBEL5* and *CalS12* were significantly down-regulated under the dual treatment (PPPS) at 4 weeks (Fig. 5).

The strongest transcriptional responses were observed under the dual treatment. *StSP6A* expression peaked at 6 weeks (2.4-fold) and again at the final harvest (2.9-fold). *StBEL5* was also up-regulated at 6 weeks (1.6-fold) under dual inoculation. In contrast, *StSP5G* showed elevated expression at both 4 and 6 weeks.

Among defence-related genes, *AOC* reached maximal expression at 6 weeks (2.1-fold) under the dual treatment. *MYC2* showed its highest expression at the final harvest (1.7-fold). *CalS12* was strongly up-regulated at 6 weeks (2.1-fold), and *ETR1* expression also increased at this time point (1.7-fold).

### Classification of expression patterns

**Fig. 6.**
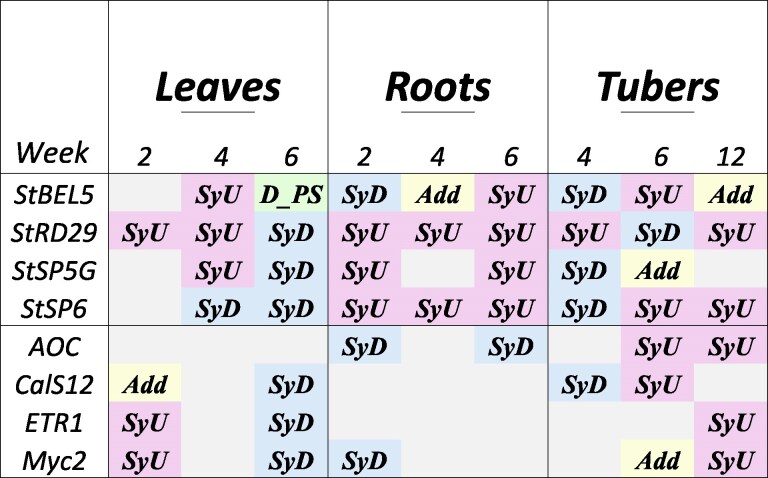
Classification of gene expression interaction types in response to bacterial inoculation, based on the data presented in Fig. 5. Each cell represents the expression pattern of an individual gene in a specific tissue at a given time point, classified according to how expression under the combined treatment (PPPS) compared with the single inoculations of *Pseudomonas protegens* CHA0 (PP) and *P. simiae* WCS417 (PS). Interaction types were assigned based on fold change comparisons and statistical analyses as follows: synergistic, where PPPS fold change significantly exceeded (SyU, red) or was lower than (SyD, blue) the mean of PP and PS by >20%; additive (Add, yellow), where PPPS fold change remained within ±5% of the mean of PP and PS; and dominant (D, green), where PPPS expression closely matched either PP or PS (within ±5%), while PP and PS differed from each other by >5%. ‘Neutral’ indicates that none of the treatments differed significantly from the control, whereas ‘Other’ denotes patterns that did not fit the defined interaction categories; both are shown in grey. Genes analysed included those associated with tuberization (*StBEL5*, *StRD29*, *StSP6A*, and *StSP6*) and defence signalling (*AOC*, *MYC2*, *ETR1*, and *CalS12*). Expression interactions are summarized across leaves, roots, and tubers at 2, 4, 6, and 12 weeks post-inoculation.

To evaluate potential interaction effects between bacterial strains, gene expression responses were classified according to whether dual inoculation produced additive, synergistic, or distinct regulatory effects compared with individual bacterial treatments (Fig. 6).

Across tissues and time points, many genes displayed non-additive responses under dual inoculation. Synergistic responses, defined as expression levels in the PPPS treatment exceeding or falling below the average of the two single-strain treatments, were observed for multiple genes. Such responses were particularly frequent for tuberization-related genes in root tissues.

Additive responses, where PPPS expression approximated the mean of PP and PS treatments, were less frequent but were observed for *StBEL5* and *MYC2* in roots and tubers. Dominant responses, in which PPPS expression resembled one of the single treatments, were also detected, particularly for *StBEL5* in leaves. Neutral responses, where expression remained similar to the control across treatments, were relatively uncommon and occurred primarily for defence-related genes.

## Discussion

Beneficial plant-associated bacteria can influence plant performance through multiple interacting mechanisms, including nutrient mobilization, hormone production, and activation of defence signalling pathways. However, the extent to which interactions among microbial strains influence these processes remains poorly understood. In this study, we investigated how two well-characterized PGPR strains, *P. protegens* CHA0 and *P. simiae* WCS417, influence potato development and signalling responses when applied individually or in combination. By integrating metabolomic profiling of bacterial exudates with analyses of plant growth and gene expression across tissues and developmental stages, our results provide insight into how microbial interactions may shape plant developmental and defence signalling networks.

### Plant growth-promoting rhizobacteria GPR functional traits and root colonisation

PGPR can influence plant development through a range of mechanisms including nutrient mobilization, hormone production, and induction of plant defence responses (Sharma *et al*., 2025b). In the present study, the two bacterial strains PP and PS exhibited several traits commonly associated with beneficial plant–microbe interactions, including IAA production, phosphate solubilization, and HCN production (Fig. 1A–C). These traits have been widely reported for plant-associated *Pseudomonas* species and are considered important contributors to their growth-promoting and biocontrol capacities (e.g. Pieterse *et al*., 2014, 2021; Waghunde and Sabalpara, 2021).

Although both strains displayed these characteristics, their relative trait profiles differed. PS showed stronger IAA production and phosphate solubilization capacity, whereas PP is widely recognized for producing antimicrobial metabolites that contribute to pathogen suppression (Gross and Loper, 2009; Ramette *et al*., 2011; Balthazar *et al*., 2022). Such differences suggest that the two strains may provide complementary functions when present together in the rhizosphere. Confocal microscopy further confirmed that both strains were able to colonize potato roots rapidly, with bacterial cells observed on root surfaces and within intercellular spaces within 24 h of inoculation (Fig. 1D). Similar colonization patterns have been reported for other beneficial *Pseudomonas* strains interacting with plant roots (Chin-A-Woeng *et al*., 1997; Duijff *et al*., 1997; Gamalero *et al*., 2004). Establishment of bacterial populations at the root surface is an important prerequisite for PGPR activity, as close spatial proximity enables chemical communication and metabolite exchange between microbes and host tissues (Venturi and Keel, 2016).

Together, these results confirm that both bacterial strains possess functional traits associated with PGPR activity and can successfully establish root associations with potato plants under the experimental conditions used in this study.

### Bacterial metabolite profiles and signalling potential

Metabolomic analyses revealed substantial differences in metabolite composition between the two bacterial strains as well as between single-strain and combined cultures (Fig. 2A–D). Many metabolites were detected predominantly in the extracellular fractions of bacterial cultures, suggesting that these compounds may participate in chemical interactions occurring in the surrounding environment.

Several amino acids detected in the supernatant fractions, including L-glutamic acid, L-glutamine, and L-arginine, may contribute to plant–microbe interactions through multiple mechanisms. In addition to serving as nitrogen sources, amino acids can act as precursors for plant hormone biosynthesis and signalling pathways that regulate root development and stress responses. For example, auxin biosynthesis pathways in both plants and microbes often utilize amino acid precursors, and auxin signalling is known to regulate root architecture and lateral root formation (Overvoorde *et al*., 2010; Zhao, 2010). In contrast, cytokinins can exert antagonistic effects on root growth (Werner *et al*., 2003). Bacterial production or modulation of hormone-related metabolites may therefore influence plant developmental responses in the rhizosphere.

Benzenoid compounds, including derivatives of SA, were also detected in bacterial supernatants. SA plays a central role in plant immune signalling and systemic acquired resistance (Vidhyasekaran, 2014; Zhang *et al*., 2025). Although the extent to which bacterial-derived SA analogues influence host signalling remains uncertain, their presence suggests that bacterial metabolites may contribute to priming or modulation of plant defence responses.

Carbohydrate-related metabolites such as glucuronic acid were also enriched in extracellular fractions. Such compounds have been associated with osmotic balance and stress tolerance in microbial systems and may contribute to microbial survival or interactions within the rhizosphere environment.

In contrast to these extracellular metabolites, fatty acids were detected more frequently in pellet fractions than in supernatants. Fatty acids are key components of bacterial membranes and are also associated with biofilm formation, which can enhance bacterial persistence on root surfaces and facilitate long-term colonization (Rieusset *et al*., 2020). Biofilm formation has been proposed as an important ecological strategy that supports stable plant–microbe interactions in the rhizosphere (Dobrzyński and Jakubowska, 2025). Together, these observations suggest that both bacterial strains produce diverse metabolites that could potentially influence plant development, defence signalling, and microbial ecological interactions.

### Metabolic interactions in the dual bacterial culture

The metabolomic data further revealed notable differences between single-strain cultures and the combined PPPS treatment. In several cases, metabolite levels in the dual culture differed substantially from those observed in either individual strain, suggesting that co-cultivation altered metabolic activity.

For example, elevated levels of metabolites such as xanthine, pipecolic acid, cAMP, FAD, and L-methionine were detected in the combined culture. Recent studies have associated similar metabolite shifts with microbial stress responses, redox regulation, and broader changes in cellular metabolism (e.g. Mhlongo *et al*., 2022). Pipecolic acid, for example, is associated with systemic resistance signalling in plants (Caddell et al., 2023), although the contribution of microbially derived pipecolic acid to plant–microbe interactions remains unclear (Sharma *et al*., 2025a).

Conversely, several intermediates of the TCA cycle were reduced in the dual bacterial culture relative to single-strain treatments. These included citric acid, isocitric acid, α-ketoglutaric acid, succinic acid, and fumaric acid. The reduction of these central metabolic intermediates may indicate changes in carbon allocation or metabolic flux when the two bacterial strains are grown together (Faust and Raes, 2012; Ravikrishnan *et al*., 2020). Alternatively, these differences could reflect differential nutrient depletion or growth dynamics between the strains during co-culture rather than direct cooperative regulation (Morris *et al*., 2013; Zelezniak *et al*., 2015).

Several metabolites, including AICAR and branched-chain amino acid intermediates such as ketoleucine and α-ketoisovaleric acid, were strongly reduced in the combined culture, which could reflect broader changes in bacterial metabolic regulation, including nucleotide metabolism and amino acid biosynthesis under co-culture conditions (Loper *et al*., 2012; Berendsen *et al*., 2015; Stringlis *et al*., 2018). Similar metabolic adjustments have been described in microbial systems where interacting species partition metabolic functions or redistribute metabolic pathways (Basan *et al*., 2015; Tsoi *et al*., 2018; Roell *et al*., 2019). However, the precise mechanisms underlying these changes remain uncertain, and further experiments would be required to determine whether they represent metabolic competition, cooperative interactions, or other regulatory processes.

Overall, the metabolomic results suggest that co-cultivation altered bacterial metabolic activity relative to single-strain cultures, potentially reflecting shifts in resource use, metabolic regulation, or inter-strain interactions.

### Effects on root development and plant growth

Inoculation with either bacterial strain, as well as their combined treatment, enhanced root development in both potato cultivars compared with uninoculated controls (Fig. 3). Treated plants exhibited increased lateral root proliferation and earlier stolon formation, indicating that bacterial inoculation may influence early developmental transitions associated with tuber initiation.

PGPR-mediated stimulation of root development has been widely reported and is often associated with bacterial production of auxins or other signalling compounds that influence root architecture (Overvoorde *et al*., 2010; Zhao, 2010). Enhanced root branching may increase the absorptive surface area of the root system, potentially improving nutrient uptake and plant growth.

Although both cultivars responded positively to bacterial inoculation, the magnitude of the response differed between cultivars. Désirée showed stronger morphological responses than ‘Mandel’, suggesting that host genotype may influence responsiveness to PGPR inoculation. Genotype-dependent responses to microbial inoculation have been documented in several plant systems and may reflect differences in root exudate composition, microbial recognition mechanisms, or hormonal regulation (Chea *et al*., 2021).

Despite clear effects on root development and above-ground growth parameters, bacterial treatments had limited influence on tuber quality traits such as starch content, soluble sugar levels, specific gravity, and ascorbic acid concentration (Fig. 4A–F). Although some trends were observed, variability among replicates reduced statistical support for strong treatment effects. This suggests that while PGPR inoculation can influence early developmental processes, effects on final tuber quality may depend on additional factors including cultivar characteristics and environmental conditions.

### Hormonal signalling and gene expression responses

Gene expression analyses revealed dynamic changes in both developmental and defence-related signalling pathways following bacterial inoculation. In particular, the strong induction of the tuberigen gene *StSP6A* in roots 2 weeks after inoculation suggests that bacterial treatments may influence early tuberization signalling pathways (Fig. 5).

*StSP6A* is a key regulator of tuber formation, and functions as a mobile signal transported from leaves to stolons where tuber initiation occurs (Navarro *et al*., 2011; Plantenga *et al*., 2019). The observed root induction of *StSP6A* shortly after bacterial inoculation may therefore reflect early developmental signalling events associated with stolon formation.

Similarly, *StBEL5*, which encodes a mobile RNA signal involved in tuberization signalling, showed increased expression in roots following bacterial treatment. Previous studies have demonstrated that *StBEL5* can regulate *StSP6A* expression and participate in long-distance signalling pathways that control tuber development (Banerjee *et al*., 2006; Sharma *et al*., 2016). These observations suggest that bacterial inoculation may influence components of the regulatory network controlling tuber initiation.

In addition to developmental regulators, several genes associated with plant defence signalling pathways were transiently induced following bacterial inoculation. Early activation of JA-related genes such as *AOC* and *MYC2* in roots may represent an initial host response to microbial perception. Beneficial microbes are known to activate signalling pathways associated with induced systemic resistance (ISR), often involving JA and ethylene signalling (Pieterse *et al*., 2014; Song *et al*., 2022).

The observed temporal changes in defence gene expression may therefore reflect an early signalling response followed by subsequent regulatory adjustments as the plant–microbe interaction stabilizes. Such dynamic transcriptional responses have been reported previously in plant–PGPR interactions and may represent part of the signalling processes that allow plants to distinguish beneficial microbes from pathogens (Stringlis *et al*., 2018).

Interestingly, the concurrent activation of tuberization-related and defence-related genes suggests that developmental and immune signalling pathways may be coordinated rather than strictly antagonistic in response to beneficial microbial interactions.

### Interaction effects between bacterial strains

Classification of gene expression responses revealed that combined bacterial inoculation frequently produced non-additive transcriptional patterns relative to single-strain treatments. In several cases, the combined PPPS treatment resulted in gene expression levels that differed from the average of the individual treatments (Fig. 6).

Such non-additive responses suggest that microbial interactions may influence plant signalling outcomes in ways that cannot be predicted solely from single-strain effects. Similar phenomena have been observed in studies of microbial consortia, where interactions among microbes can modify metabolite production, signalling processes, and ecological behaviour (Berendsen *et al*., 2018; Roell *et al*., 2019).

Although the mechanistic basis of these interactions remains uncertain, several potential explanations exist. Bacterial strains may alter each other’s metabolic activity through competition for resources, cross-feeding of metabolites, or modulation of signalling pathways. Such interactions could lead to the production of metabolite combinations that differ from those produced by either strain alone, thereby influencing plant responses.

Overall, the results suggest that microbial consortia may generate complex signalling environments that influence plant developmental and defence pathways.

### Limitations and future perspectives

While the present study provides insights into PGPR-mediated signalling responses in potato, several limitations should be considered when interpreting the results. The experiments were conducted under controlled greenhouse conditions using defined bacterial inoculations and sterile plant material. Such experimental systems allow mechanistic investigation of plant–microbe interactions but will not represent the complexity of agricultural soils, where diverse microbial communities, environmental fluctuations, and soil physicochemical properties can influence plant responses.

Consequently, the magnitude and stability of the observed effects may differ under field conditions. Future studies should therefore evaluate whether similar interactions are maintained in more complex soil environments and whether microbial consortia produce similar effects under agricultural management conditions.

Further work will also be required to determine the specific molecular mechanisms linking bacterial metabolites to plant developmental signalling pathways. Integrating metabolomics, transcriptomics, and functional genetic approaches may help clarify the signalling processes underlying PGPR-mediated growth promotion.

## Conclusion

**Fig. 7.**
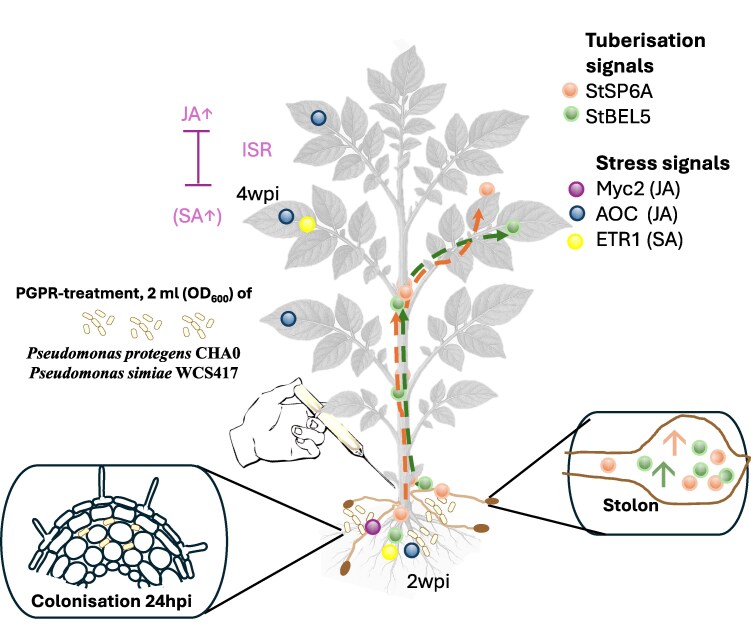
Model for tuber induction in PGPR-inoculated potato plants under long-day conditions. Under 16 h light/8 h dark conditions, potato roots inoculated with *Pseudomonas protegens* CHA0 (PP) and *P. simiae* WCS417 (PS) show early root colonization within 24 hours post-inoculation (24 hpi). By 2 weeks post-inoculation (2 wpi), below-ground (BG) root–microbe interactions trigger systemic signalling to above-ground (AG) tissues, promoting tuber induction. Key mobile signals such as StSP6A and StBEL5 are activated in roots and leaves and transported to stolons, where they promote tuber initiation. This process is tightly regulated by hormonal signalling: JA-associated signalling responses are observed in roots, while SA-related responses appear reduced at certain time points, potentially creating a physiological context that may favour tuber formation. The JA-responsive transcription factor MYC2 and ethylene signalling via ETR1 further contribute to the coordination of induced systemic resistance (ISR) and tuberization. At 4 wpi, elevated expression of *AOC* (a JA biosynthesis gene) is observed in leaves in response to all treatments, while *CalS12* and *ETR1* are specifically up-regulated in response to PP treatment only.

In summary, this study demonstrates that the PGPR strains *P. protegens* CHA0 and *P. simiae* WCS417 influence potato development and signalling responses through multiple interacting mechanisms (Fig. 7). The two strains exhibit distinct metabolic profiles and complementary functional traits, and their combined application frequently resulted in non-additive transcriptional responses in the host plant. Although the mechanistic links between bacterial metabolites and plant signalling remain to be fully resolved, the results suggest that microbial interactions may influence plant developmental and defence pathways in ways not predictable from single-strain inoculations alone. These findings highlight the potential importance of microbial consortia in shaping plant performance, and provide a foundation for future research exploring PGPR-based strategies for sustainable crop production. To our knowledge, this study represents one of the first attempts to integrate PGPR metabolite profiling with spatiotemporal analyses of tuberization signalling in potato.

## Supplementary Material

erag237_Supplementary_Data

## Data Availability

All data supporting the findings of this study are available within the paper and its supplementary data published online.

## Supplementary data

The following supplementary data are available at *JXB* online.

Dataset S1. Excel file summarizing the gene expression analyses, including separate sheets for primer information, raw expression data, and fold change significance analyses.

Dataset S2. Excel file summarizing the metabolomics analyses, including separate sheets containing LC-MS raw data and heatmap-associated datasets.
